# Reviewers in 2022

**DOI:** 10.1098/rsif.2023.0078

**Published:** 2023-03-15

**Authors:** Richard Cogdell

**Affiliations:** Editor-in-Chief

*Editor-in-Chief*

I thank all those referees who helped assess submissions to *Journal of the Royal Society Interface* (volume 19) in 2022. The editorial team very much appreciates the time and dedication given by academics to the peer review process and recognizes their efforts. From previous feedback, we know that most reviewers value recognition of their efforts by the journal and through services, such as Publons, which is integrated with *J. R. Soc. Interface*.

To provide an opportunity for recognition, we list the names of all reviewers who provided reports last year (unless they have opted out or not responded). This article is made permanent and citable by its digital object identifier (DOI). We encourage you to quote your contribution in your applications for tenure and grants or other forms of research assessment. Where you have provided it, we have included your ORCID so your contribution can be unambiguously assigned to you.

The team really do appreciate all the work our reviewers do on behalf of the journal, and we look forward to working with you again in 2023.

Abbass H
Abbey B
Abe T
Åberg C
Ackerman A
Adam I
Adams P
Adouni M
Aflalo C https://orcid.org/0000-0002-3864-0666
Agostinelli D
Agrawal D
Ahator SD
Alampounti A
Alburez-Gutierrez Dhttps://orcid.org/0000-0002-9823-5179
Alegana Vhttps://orcid.org/0000-0001-5177-9227
Alessandretti L
Alexov E
Alshareef A
Altmann Ehttps://orcid.org/0000-0002-1932-3710
Amlani Fhttps://orcid.org/0000-0003-4022-8088
an der Heiden M
Anderson C
Anderson M
Anderson Phttps://orcid.org/0000-0001-7133-8322
Andersson E
Ando N
Andraud M
Androulakis I
Ansell B
Aono Hhttps://orcid.org/0000-0003-2427-0833
Araujo-Estrada S
Arzani Ahttps://orcid.org/0000-0002-3706-7909
Ashmore J
Auth T
Avazmohammadi Rhttps://orcid.org/0000-0001-9787-1117
Avgar Thttps://orcid.org/0000-0002-8764-6976
Avital E
Backholm M
Baines Chttps://orcid.org/0000-0002-6918-3648
Balabani Shttps://orcid.org/0000-0002-6287-1106
Bálint Z
Balmain A
Banerjee S
Banga JRhttps://orcid.org/0000-0002-4245-0320
Banker Rhttps://orcid.org/0000-0001-5020-6670
Banzhaf W
Barakat Ahttps://orcid.org/0000-0002-7551-3631
Bardwell L
Baron Jhttps://orcid.org/0000-0003-2815-3263
Bartlett Mhttps://orcid.org/0000-0002-7391-5135
Bartumeus Fhttps://orcid.org/0000-0001-6908-3797
Bastard Jhttps://orcid.org/0000-0001-8531-1614
Bauch Chttps://orcid.org/0000-0001-6214-6601
Beatus Thttps://orcid.org/0000-0002-9875-2766
Beckman R
Bekiranov S
Bellomo N
Benítez Mhttps://orcid.org/0000-0002-4901-2833
Benkő Z
Bennett M
Ben-Nun M
Berbert J
Bertram J
Bertrand Ohttps://orcid.org/0000-0002-0889-4550
Beskos A
Betta M
Bhagat Rhttps://orcid.org/0000-0002-8928-4534
Bhatia S
Bhattacharya Khttps://orcid.org/0000-0002-4943-3814
Bhavnani D
Bianconi G
Bienfait A
Biga Vhttps://orcid.org/0000-0001-9592-385X
Binder W
Biswas Dhttps://orcid.org/0000-0003-4141-4034
Blanch-Mercader C
Blinder P
Blüthgen Nhttps://orcid.org/0000-0002-0171-7447
Bodzioch Mhttps://orcid.org/0000-0002-1991-5062
Boiani M
Bonasoro F
Boos C
Booth D
Borowska A
Boyer Dhttps://orcid.org/0000-0003-2676-6752
Bravi B
Brembs Bhttps://orcid.org/0000-0001-7824-7650
Breto C
Breuer K
Britton Thttps://orcid.org/0000-0002-9228-7357
Brook B
Brooks L
Brown A
Brown S
Brownell W
Bruijn Shttps://orcid.org/0000-0003-0290-2131
Brunner Jhttps://orcid.org/0000-0002-8147-2522
Bulens D
Bull Jhttps://orcid.org/0000-0003-1475-2898
Bursa J
Byrnes G
Cadiou H
Cañal-Bruland R
Caponi S
Capraro Vhttps://orcid.org/0000-0002-0579-0166
Carbone G
Cardini Ahttps://orcid.org/0000-0003-2910-632X
Carré M
Casas Jhttps://orcid.org/0000-0003-1666-295X
Catalán Phttps://orcid.org/0000-0003-2826-4684
Catoni Mhttps://orcid.org/0000-0002-3258-2522
Chamorro L
Champredon D
Chandran Suja Vhttps://orcid.org/0000-0002-3406-7655
Chapman Jhttps://orcid.org/0000-0002-7475-4441
Checa Ahttps://orcid.org/0000-0001-7873-7545
Chechkin A
Chen J
Chen Y
Cheney Jhttps://orcid.org/0000-0002-9952-2612
Cheong KHhttps://orcid.org/0000-0002-4475-5451
Chesler NC
Chevalier Nhttps://orcid.org/0000-0002-9713-1511
Chico T
Chinappi M
Cho H
Chraibi M
Christians J
Ciaccio E
Cinquemani E
Claessen B
Clemente Chttps://orcid.org/0000-0001-8174-3890
Coccia M
Codding Bhttps://orcid.org/0000-0001-7977-8568
Colnaghi M
Conlan Ahttps://orcid.org/0000-0002-2593-6353
Connizzo B
Conradi C
Convertino Mhttps://orcid.org/0000-0001-7003-7587
Conway Jhttps://orcid.org/0000-0003-1474-4230
Cool L
Corbat Ahttps://orcid.org/0000-0001-8068-3486
Cornuault P-H
Courau T
Cowan N
Crauste F
Cribellier Ahttps://orcid.org/0000-0001-5113-7531
Cutsforth-Gregory Jhttps://orcid.org/0000-0002-5689-4424
Czosnyka Z
Dabagh Mhttps://orcid.org/0000-0003-1540-7420
Dal Corso Fhttps://orcid.org/0000-0001-9045-8418
D'Alba Lhttps://orcid.org/0000-0002-2478-3455
Davies P
de Jong Hhttps://orcid.org/0000-0002-2226-650X
de Jong Mhttps://orcid.org/0000-0002-5339-1995
de los Reyes VAhttps://orcid.org/0000-0001-5418-4579
De Luca P
Debarre Fhttps://orcid.org/0000-0003-2497-833X
Delgado Chaves F
Delhaye Bhttps://orcid.org/0000-0003-3974-7921
Delitala M
Demers Jhttps://orcid.org/0000-0002-8019-4153
Dentz Mhttps://orcid.org/0000-0002-3940-282X
Derégnaucourt S
Deziel E
Di Stefano A
Dickerson B
Dickinson G
Dingli D
Ditlevsen P
Dolson Ehttps://orcid.org/0000-0001-8616-4898
Domenech de Cellès Mhttps://orcid.org/0000-0002-9302-4858
Donatelli C
Drake A
Duan Y
Dunlop J
Durand Bhttps://orcid.org/0000-0003-0669-1394
Dushoff Jhttps://orcid.org/0000-0003-0506-4794
Dutta Phttps://orcid.org/0000-0001-6067-1023
Dymond Mhttps://orcid.org/0000-0002-9903-4993
Edwards A
Elliott Khttps://orcid.org/0000-0001-5304-3993
Ellison N
Eloy C
Emani Phttps://orcid.org/0000-0002-3091-5231
Ene-Iordache Bhttps://orcid.org/0000-0002-4011-9343
Engelstaedter Jhttps://orcid.org/0000-0003-3340-918X
Epari D
Érdi P
Errington Thttps://orcid.org/0000-0002-4959-5143
Espino D
Evans S
Farid Mhttps://orcid.org/0000-0002-6734-9931
Fadikar Ahttps://orcid.org/0000-0001-7396-0350
Farris Dhttps://orcid.org/0000-0002-6720-1961
Federle M
Figueiredo S
Finlayson Ehttps://orcid.org/0000-0002-0433-5313
Fouda M
Fox-Kemper Bhttps://orcid.org/0000-0002-2871-2048
Franco Ehttps://orcid.org/0000-0003-1103-2668
Frank M
Franklin D
Franzese G
Frasca P
Frei T
Fried S
Friedman Mhttps://orcid.org/0000-0001-8279-0306
Friedrich B
Friston Khttps://orcid.org/0000-0001-7984-8909
Fromen Chttps://orcid.org/0000-0002-7528-0997
Fruleux Ahttps://orcid.org/0000-0003-2974-3337
Fu B
Fuhrmann-Lieker Thttps://orcid.org/0000-0003-3473-534X
Gadhoumi K
Gallos L
García Guirao JL
Garcia-Aznar J
Garcia-Martin JAhttps://orcid.org/0000-0003-0993-4064
Garira W
Garnier Shttps://orcid.org/0000-0002-3886-3974
Gaucherel C
Geard Nhttps://orcid.org/0000-0003-0069-2281
Geitmann A
Genin Ghttps://orcid.org/0000-0003-3612-4729
Georgiades M
Gerringer M
Gerstein Ahttps://orcid.org/0000-0002-0781-9356
Ghajari Mhttps://orcid.org/0000-0001-5678-5124
Ghandiali Shttps://orcid.org/0000-0002-6845-774X
Gholami Ahttps://orcid.org/0000-0002-9332-4551
Ghomlaghi M
Gil JP
Gilan O
Gilet Thttps://orcid.org/0000-0002-7339-7796
Girard M
Gohain Barua Ahttps://orcid.org/0000-0001-6724-4390
Golchin Mhttps://orcid.org/0000-0002-5288-0121
Gómez-Nava L
Gompper G
Gonzalez-Parra Ghttps://orcid.org/0000-0001-5847-678X
Goodman J
Gopinath Ahttps://orcid.org/0000-0002-7824-3163
Gorbunov M
Gordon M
Gordus A
Goris R
Gorzelak Phttps://orcid.org/0000-0001-5706-1881
Gosler A
Gosselin F
Grabowski Ahttps://orcid.org/0000-0002-4432-618X
Graf Ahttps://orcid.org/0000-0003-4870-7622
Grant A
Gray K
Grebenkov Dhttps://orcid.org/0000-0002-6273-9164
Griffen Bhttps://orcid.org/0000-0002-8126-6323
Grima Rhttps://orcid.org/0000-0002-1266-8169
Groom D
Gruber P
Gubbins Shttps://orcid.org/0000-0003-0538-4173
Gueorguiev D
Guerrero P
Guillon V
Guimapi R
Guo Bhttps://orcid.org/0000-0002-6157-7183
Gupta A
Gurarie Dhttps://orcid.org/0000-0002-5314-7888
Guttal V
Haaland T
Haider N
Hall Ihttps://orcid.org/0000-0002-3033-2335
Hamelin Fhttps://orcid.org/0000-0003-2653-699X
Hamilton Mhttps://orcid.org/0000-0003-1754-6969
Han TAhttps://orcid.org/0000-0002-3095-7714
Hare Phttps://orcid.org/0000-0001-5014-6161
Harries K
Hartmann Mhttps://orcid.org/0000-0001-6046-0365
Hatala K
Hatna E
Havlicek V
Hawdon JM
He Dhttps://orcid.org/0000-0003-3253-654X
Hedwig Bhttps://orcid.org/0000-0002-1132-0056
Heeney M
Hein J
Held N
Hellmich C
Henderson Ahttps://orcid.org/0000-0001-9233-5059
Henningsson Phttps://orcid.org/0000-0003-2640-1067
Herbst Ehttps://orcid.org/0000-0003-3640-9695
Hickson R
Hills T
Hiscock T
Hladish TJ
Ho S-H
Hoi Hhttps://orcid.org/0000-0002-3038-9673
Hoinville Thttps://orcid.org/0000-0001-9073-4515
Holder Chttps://orcid.org/0000-0002-9149-8996
Holle A
Holowka Nhttps://orcid.org/0000-0003-0593-7524
Holten-Andersen N
Hossain MS
Howell L
Hoyle A
Hoyle R
Hsieh T
Hu Dhttps://orcid.org/0000-0002-0017-7303
Hu K
Huettmann F
Hulsey CDhttps://orcid.org/0000-0002-9653-6728
Iaparov Bhttps://orcid.org/0000-0002-3398-2093
Ibanez-Guzman J
Ikeda K
Iliff J
Imai H
Imhof L
Ingalls B
Irbäck A
Islam MR
Issah I
Jackson J
Jacobberger J
Jacobson Ahttps://orcid.org/0000-0002-5661-3821
Jaganathan GKhttps://orcid.org/0000-0003-0972-9263
Jain Hhttps://orcid.org/0000-0002-9662-0444
Jankauski Mhttps://orcid.org/0000-0001-6305-0564
Jansson Fhttps://orcid.org/0000-0001-8357-0276
Jaqaman K
Jashnsaz H
Jensen Ohttps://orcid.org/0000-0003-0172-6578
Ji S
Johansson Chttps://orcid.org/0000-0002-1851-3635
Johnson E
Jones A
Jones E
Jones L
Jong ICd
Junttila Shttps://orcid.org/0000-0001-8276-9259
Jurisch-Yaksi Nhttps://orcid.org/0000-0002-8767-6120
Kadish Dhttps://orcid.org/0000-0003-3573-2917
Kaimaki D-Mhttps://orcid.org/0000-0002-6104-1442
Kainz M
Kandil K
Kang Vhttps://orcid.org/0000-0003-0959-1364
Kangas J
Kasper Rhttps://orcid.org/0000-0002-3457-1931
Katzner Thttps://orcid.org/0000-0003-4503-8435
Kelly S
Kenah Ehttps://orcid.org/0000-0002-7117-7773
Kennedy WG
Kenney L
Kenny Mhttps://orcid.org/0000-0002-6824-9377
Ketcheson D
Keyel Ahttps://orcid.org/0000-0001-5256-6274
Khain Ehttps://orcid.org/0000-0002-4631-836X
Khaluf Yhttps://orcid.org/0000-0002-5590-9321
King Hhttps://orcid.org/0000-0003-3299-5690
King M
Kitagawa H
Kleiven S
Klumpp S
Knight Ghttps://orcid.org/0000-0002-7263-9896
Koch Thttps://orcid.org/0000-0003-4776-5222
Köhn-Luque A
Kokash Nhttps://orcid.org/0000-0003-3639-1245
Kolomenskiy Dhttps://orcid.org/0000-0003-0107-6894
Kornev Khttps://orcid.org/0000-0002-4513-1915
Kostylev M
Kraikivski P
Kratochvilova I
Krauland M
Kremling A
Kretzberg J
Krishnan Jhttps://orcid.org/0000-0001-6196-2033
Krishnan Lhttps://orcid.org/0000-0002-3519-5341
Kuchenbecker Khttps://orcid.org/0000-0002-5004-0313
Kumar A
Kumar P
Kunaal J
Kurasaki M
Kurian Phttp://orcid.org/0000-0002-4160-6434
Kurniawan N
Kwiatkowska D
Labouta H
Labra-Vázquez P
Lacquaniti F
Lafferty Khttps://orcid.org/0000-0001-7583-4593
Lagergren J
Lai Y-Chttps://orcid.org/0000-0003-0801-8830
Lamata Phttps://orcid.org/0000-0002-3097-4928
Lancia L
Langeslay B
Laradji M
Larkum A
Larsen PShttps://orcid.org/0000-0002-7155-5965
Laubrie J
Laurino M
Lautenschlager Shttps://orcid.org/0000-0003-3472-814X
Lazebnik T
Le Rouzic Ahttps://orcid.org/0000-0002-2158-3458
Leander Rhttps://orcid.org/0000-0002-1392-9754
Lecheval V
Lee DS
Lefèvre P
Lega Jhttps://orcid.org/0000-0003-2064-229X
Legallais C
Lehmann Lhttps://orcid.org/0000-0001-9549-9577
Lemaire K
Lenormand Mhttps://orcid.org/0000-0001-6362-3473
Leon Thttps://orcid.org/0000-0002-7633-677X
Lerma C
Lerma-Hernandez SA
Levien Ehttps://orcid.org/0000-0001-6825-2966
Levinson Dhttps://orcid.org/0000-0002-4563-2963
Lewandowska K
Li H-W
Li Lhttps://orcid.org/0000-0002-6741-9741
Li W
Liechti Fhttps://orcid.org/0000-0001-9473-0837
Lilleri D
Limjeerajarus Nhttps://orcid.org/0000-0001-5007-9165
Lin J
Lingam Mhttps://orcid.org/0000-0002-2685-9417
Linhart P
Linkov I
Lisicki M
Liu Bhttps://orcid.org/0000-0002-2965-7714
Liu Jhttp://orcid.org/0000-0002-7445-3518
Liu Yhttps://orcid.org/0000-0003-2640-6490
Loder A
Loman Bhttps://orcid.org/0000-0001-8830-7506
Lombardi F
Long Y
Lövkvist C
Lu Jhttps://orcid.org/0000-0001-5753-5362
Lucassen P
Lürig Mhttps://orcid.org/0000-0002-8175-6234
Lyutova Rhttps://orcid.org/0000-0002-4922-2785
M. Wood C
Macnamara C
Madera R
Madhusudhana Shttps://orcid.org/0000-0002-4142-3881
Magpantay Fhttps://orcid.org/0000-0002-1064-0357
Maier C
Mandal S
Mangkalaphiban K
Manhart A
Manikandan MS
Manley E
Marchisio M
Marcotti S
Marini Ghttps://orcid.org/0000-0001-9721-7211
Marino J
Markov D
Marsland Shttps://orcid.org/0000-0002-9568-848X
Martelli Chttps://orcid.org/0000-0002-5663-6580
Martino C
Martin O
Martins J
Marxer R
Matijevich E
Matrajt L
Mauksch M
Mayalu M
Mazzoli Mhttps://orcid.org/0000-0002-8756-5535
Mbaeyi C
McCarthy M
McCarthy Khttps://orcid.org/0000-0002-9148-8448
McCoy Dhttps://orcid.org/0000-0001-8383-8084
McCullough Jhttps://orcid.org/0000-0002-9606-0408
McDonald K
McFadden Jhttps://orcid.org/0000-0003-2145-0046
McGinty Shttps://orcid.org/0000-0002-2428-2669
McHenry Mhttps://orcid.org/0000-0001-5834-674X
McKeegan P
McMeeking R
Meaud J
Medina Cruz D
Meehan Mhttps://orcid.org/0000-0003-1332-332X
Mehandia V
Menci Mhttps://orcid.org/0000-0002-8889-623X
Metzler Hhttps://orcid.org/0000-0002-8239-1601
Metzler Rhttps://orcid.org/0000-0002-6013-7020
Meyer CA
Michaelian K
Mideo N
Milligan Khttps://orcid.org/0000-0001-7089-9304
Minas Ghttps://orcid.org/0000-0001-7953-706X
Mirkhalaf Mhttps://orcid.org/0000-0002-4142-6097
Mitra Bhttps://orcid.org/0000-0002-6617-0884
Mognetti B
Moiseff A
Mok W
Mongeau J-Mhttps://orcid.org/0000-0002-3292-6911
Montgomerie Rhttps://orcid.org/0000-0003-4701-4525
Monzem S
Moore S
Morbiducci Uhttps://orcid.org/0000-0002-9854-1619
Moreno AJ
Mueller B
Müller R
Munasinghe M
Mundt CC
Murawaki Y
Murray A
Myrgiotis V
Nabawy Mhttps://orcid.org/0000-0002-4252-1635
Nakao Hhttps://orcid.org/0000-0003-3394-0392
Nakata Thttps://orcid.org/0000-0002-0378-2242
Navajas Jhttps://orcid.org/0000-0001-8765-037X
Nax H
Nayak A
Neal P
Neuriter N
Nguyen D
Nguyen L
Niewiarowski P
Nikishova A
Ning N
Nixon E
Novak D
Nuckols R
Obeng-Odoom F
Obrist D
Oettmeier C
Oidtman R
Oiwa K
Okada I
Olsen Rhttps://orcid.org/0000-0002-2918-4152
Omelon S
O'Neill Mhttps://orcid.org/0000-0001-9614-7813
Opatowski Lhttps://orcid.org/0000-0001-8348-700X
Ouellet-Plamondon C
Özden S
Pagnacco M
Pain Rhttps://orcid.org/0000-0002-5354-6301
Palavalli-Nettimi Rhttps://orcid.org/0000-0002-4523-8245
Paldi A
Palmer Rhttps://orcid.org/0000-0001-8964-9014
Palombo F
Pan M
Pandith A
Papageorgiou D
Papangelo Ahttps://orcid.org/0000-0002-0214-904X
Pardo-Pastor Chttps://orcid.org/0000-0003-4083-0511
Parolini N
Parry Mhttps://orcid.org/0000-0002-6588-0219
Passerini T
Pastore Dhttps://orcid.org/0000-0002-0905-0085
Patten M
Payne S
Peixoto Phttps://orcid.org/0000-0003-2358-3221
Pekkan Khttps://orcid.org/0000-0001-7637-4445
Peleg Ohttps://orcid.org/0000-0001-9481-7967
Penzel T
Pereira CC
Perkins M
Perovic Shttps://orcid.org/0000-0003-4393-4105
Perrino Ghttps://orcid.org/0000-0001-8095-9139
Perumal A
Peters K
Petri Ghttps://orcid.org/0000-0003-1847-5031
Pfeuty Bhttps://orcid.org/0000-0002-9202-3941
Phillips B
Phillips J
Piarroux R
Picardi S
Pietruszka Mhttps://orcid.org/0000-0001-5852-9095
Pigolotti S
Pinheiro PGhttps://orcid.org/0000-0003-2397-6463
Pires R
Pisor Ahttps://orcid.org/0000-0001-5780-4542
Pitsillides Ahttps://orcid.org/0000-0002-3861-998X
Pleimling M
Plenz D
Ponce de León M
Pons A
Pons-Salort M
Pooley Chttps://orcid.org/0000-0002-8779-4477
Poon A
Porro L
Porter M
Potgieter P
Prada F
Prakash V
Prasad Ahttps://orcid.org/0000-0002-0832-8153
Priede Ihttps://orcid.org/0000-0002-5064-9751
Prince R
Pujo-Menjouet L
Purwidyantri A
Puthoff J
Putman Nhttps://orcid.org/0000-0001-8485-7455
Qiao M
Raffa Vhttps://orcid.org/0000-0002-4289-9937
Rainbow M
Raj D
Rajan Rhttps://orcid.org/0000-0002-7770-3711
Ramírez Ávila Mhttps://orcid.org/0000-0003-4522-9012
Rammer W
Rappel W-Jhttps://orcid.org/0000-0003-3833-7197
Ratcliff Whttps://orcid.org/0000-0002-6837-8355
Ray E
Rayfield E
Read Jhttps://orcid.org/0000-0002-9697-0962
Rebora Mhttps://orcid.org/0000-0002-4271-6336
Reconditi M
Reeves Dhttps://orcid.org/0000-0001-5684-9538
Rennie M
Reppert Mhttps://orcid.org/0000-0002-7390-3652
Rezgui D
Ribak G
Ribeiro Fhttps://orcid.org/0000-0002-2719-6061
Riede Thttps://orcid.org/0000-0001-6875-0017
Rienmüller Thttps://orcid.org/0000-0002-9623-106X
Rimbaud L
Riveline D
Roberts Mhttps://orcid.org/0000-0003-2693-5093
Robertson DJ
Rockne Rhttps://orcid.org/0000-0002-1557-159X
Rode C
Rodrigo Ghttps://orcid.org/0000-0002-1871-9617
Rodrigues M
Rodríguez Argüelles MC
Rodríguez J
Roh S Woon
Römhild R
Routier A-L
Roy A
Rudorf S
Ruess Jhttps://orcid.org/0000-0003-1615-3282
Ruocco G
Rychtar Jhttps://orcid.org/0000-0001-6600-2939
Saal Hhttps://orcid.org/0000-0002-7544-0196
Sacco Phttps://orcid.org/0000-0002-5559-2889
Safaei S
Saha S
Sahasranaman Ahttps://orcid.org/0000-0002-9613-8041
Saito K
Salje H
Salvioli Mhttps://orcid.org/0000-0002-7686-2753
Samee MDAH
Sánchez JÁ
Sandev T
Sankey DWEhttps://orcid.org/0000-0002-6363-8023
Santi P
Santillan Mhttps://orcid.org/0000-0001-8605-7451
Santon Mhttps://orcid.org/0000-0002-9397-4052
Sapudom J
Saracco F
Saranathan Vhttps://orcid.org/0000-0003-4058-5093
Schadschneider A
Schiavazzi D
Schlusser N
Schmal Chttps://orcid.org/0000-0002-9213-3868
Schmitt D
Schmitt Shttps://orcid.org/0000-0002-7768-8961
Schofield A
Schroeder T
Scott Jhttps://orcid.org/0000-0003-2971-7673
Scott M
Shahrezaei V
Shaman J
Shan Y
Shen S-Fhttps://orcid.org/0000-0002-0631-6343
Shepherd Jhttps://orcid.org/0000-0002-3790-0323
Shera C
Shibasaki Shttps://orcid.org/0000-0002-8196-0745
Shirtcliffe N
Shiu Y
Shrivastava A
Sieber Jhttps://orcid.org/0000-0002-9558-1324
Sierra Chttps://orcid.org/0000-0003-0009-4169
Silva CJ
Singh M
Singh P
Singh Vhttps://orcid.org/0000-0001-9391-5901
Skinner M
Smith SMhttps://orcid.org/0000-0002-7480-3581
Sodt A
Sokolis D
Sommer G
Sone E
Spedding Ghttp://orcid.org/0000-0003-3033-7897
Spencer N
Spill F
Spitzen Jhttps://orcid.org/0000-0003-0455-7511
Spool Jhttps://orcid.org/0000-0002-9130-9526
Springborn Mhttps://orcid.org/0000-0003-3476-8758
Squires Ahttps://orcid.org/0000-0002-2417-1432
Sridharan Vhttps://orcid.org/0000-0003-1457-6900
Stan G-B
Stayton T
Steel H
Steinmann Thttps://orcid.org/0000-0002-2013-7747
Stelling Mhttps://orcid.org/0000-0003-2795-2581
Stenroth L
Stone C
Strauch C
Strychalski W
Stubbs Chttps://orcid.org/0000-0002-3121-3223
Sturla Fhttps://orcid.org/0000-0001-7317-304X
Su Q
Suki Bhttps://orcid.org/0000-0002-9720-1006
Sulc P
Suzuki R
Számadó Shttps://orcid.org/0000-0003-2204-9705
Szorkovszky Ahttps://orcid.org/0000-0001-9331-3214
Taboada P
Takeda M
Tamm Khttps://orcid.org/0000-0002-2455-8258
Tanaka Mhttps://orcid.org/0000-0003-2198-1402
Tanaka Y
Tang H
Tapia Muñoz NEhttps://orcid.org/0000-0003-0018-2492
Taylor J
Teo SKhttp://orcid.org/0000-0001-5659-3763
Testori Mhttps://orcid.org/0000-0001-7292-7129
Thomas Phttps://orcid.org/0000-0003-4919-8452
Thompson Jhttps://orcid.org/0000-0003-3485-172X
Thompson K
Thompson M
Thomson R
Thurner P
Tilman A
Timashev P
Timerman Dhttps://orcid.org/0000-0002-8772-6271
Tizzoni Mhttps://orcid.org/0000-0001-7246-2341
Tobalske B
Tonković Ž
Toxvaerd F
Traulsen Ahttps://orcid.org/0000-0002-0669-5267
Tribastone M
Trzaskowska M
Turunen M
Tytell E
Uyeno T
Vaidya NK
Valdespino Q
van Belzen Jhttps://orcid.org/0000-0003-2099-1545
Vardar Yhttps://orcid.org/0000-0003-2156-1504
Verstrepen K
Villani M
Villaverde Ahttps://orcid.org/0000-0001-7401-7380
Villinger J
Vitas M
Vlach Jhttps://orcid.org/0000-0002-0874-2055
Voigt Dhttps://orcid.org/0000-0003-2772-8504
Volpert V
von der Haar Thttps://orcid.org/0000-0002-6031-9254
Voronina L
Wacker C
Waclaw B
Wade M
Wagshul M
Walani N
Waldherr Shttps://orcid.org/0000-0002-0936-579X
Waller Lhttps://orcid.org/0000-0001-5002-8886
Wan L
Wang B
Wang VYhttps://orcid.org/0000-0003-0895-3132
Wang Xhttps://orcid.org/0000-0002-9055-1011
Wang Z
Wardenaar K
Wardley W
Watanebe N
Wegener Mhttps://orcid.org/0000-0002-1076-4587
Wegst UGKhttps://orcid.org/0000-0002-9057-415X
Weickenmeier J
Weinans Ehttps://orcid.org/0000-0001-6423-9510
Weiss J
Werth Ahttps://orcid.org/0000-0002-7777-478X
Willemet Lhttps://orcid.org/0000-0002-4797-1010
Williams H
Williams-Hatala EM
Willinger R
Wilts Bhttps://orcid.org/0000-0002-2727-7128
Winfield Ahttps://orcid.org/0000-0002-1476-3127
Winters Jhttps://orcid.org/0000-0003-2982-2991
Wolf Lhttps://orcid.org/0000-0003-0274-599X
Wood R
Woodward J
Woolley T
Wu Ahttps://orcid.org/0000-0002-1801-5716
Wu JH
Wysokowski M
Xavier Jhttps://orcid.org/0000-0001-9242-8968
Xiao Fhttps://orcid.org/0000-0002-5001-5644
Yadav P
Yamamoto T
Yamazaki Shttps://orcid.org/0000-0002-6691-1531
Yang K-C
Yang Qhttps://orcid.org/0000-0003-3215-3645
Yeomans Jhttps://orcid.org/0000-0001-8268-5469
Yi H
Yoshihara Mhttps://orcid.org/0000-0002-8915-9282
Young Jhttps://orcid.org/0000-0002-8671-990X
Youssofzadeh V
Yu P Yhttps://orcid.org/0000-0003-2406-8723
Yunker P
Zachos L
Zachreson Chttps://orcid.org/0000-0002-0578-4049
Zadeh-Haghighi Hhttps://orcid.org/0000-0003-3380-9925
Zahradka D
Zahradníková A
Zajdel Thttps://orcid.org/0000-0002-7957-3180
Zamponi Fhttps://orcid.org/0000-0001-9260-1951
zaslansky phttps://orcid.org/0000-0002-8714-4992
Zavodszky Ghttps://orcid.org/0000-0003-0150-0229
Zelner Jhttps://orcid.org/0000-0002-0708-2008
Zenit Rhttps://orcid.org/0000-0002-2717-4954
Zhang F
Zhang Jhttps://orcid.org/0000-0001-7107-4814
Zhao H
Zhdanov O
Zheng Xhttps://orcid.org/0000-0003-4993-1896
Zuluaga M
Zuriguel I

